# Exploring CACNA1H as a potential candidate biomarker for calcific aortic valve disease

**DOI:** 10.1016/j.isci.2026.115616

**Published:** 2026-04-27

**Authors:** Jun Chen, Liusheng Wang, Wenyan Li, Shunyi Li, Xiaolin Duan, Sijie Jiang, Zhen Zhang, Qingchun Zeng

**Affiliations:** 1 State Key Laboratory of Organ Failure Research, Department of Cardiology, Nanfang Hospital, Southern Medical University, Guangzhou 510515, China; 2 Guangdong Provincial Key Laboratory of Shock and Microcirculation, Southern Medical University, Guangzhou 510515, China; 3 Guangdong Cardiovascular Institute, Guangdong Provincial People’s Hospital, Guangdong Academy of Medical Sciences, Southern Medical University, Guangzhou, Guangdong, China

**Keywords:** Health sciences, Medicine, Medical specialty, Internal medicine, Cardiovascular medicine

## Abstract

Calcific aortic valve disease (CAVD) is a prevalent cardiovascular disorder characterized by calcium deposition in the aortic valve, associated with high morbidity and mortality. Mechanical stress plays a key role in its pathogenesis, highlighting the importance of identifying mechanosensitive ion channel-related genes (MICRGs). In this study, transcriptome data from GSE83453 of patients with CAVD and healthy controls were analyzed to identify differentially expressed genes (DEGs). Weighted gene co-expression network analysis (WGCNA) and MICRGs were used to pinpoint key genes. The intersection of DEGs, WGCNA modules, and MICRGs identified *CACNA1H* as a candidate gene. External validation with GSE51472 and GSE55492 confirmed these findings. Both *in vitro* and *in vivo* studies confirmed that CACNA1H inhibition alleviates CAVD progression by repressing the osteogenic response. Mechanistically, CACNA1H inhibition attenuated phosphorylation of P65, a key regulator of the NF-κB pathway. These results suggest that *CACNA1H* may serve as a promising biomarker and therapeutic target for CAVD.

## Highlights

• *CACNA1H* identified as a potential biomarker and correlates with TLR and NF-κB pathways in CAVD
• *CACNA1H* is upregulated in CAVD and promotes osteogenesis and valve calcification
• *CACNA1H* modulates inflammation and osteogenic differentiation through NF-κB/p65
• Inhibition of CACNA1H ameliorates aortic valve calcification *in vivo*

## Graphical abstract

**Figure:**
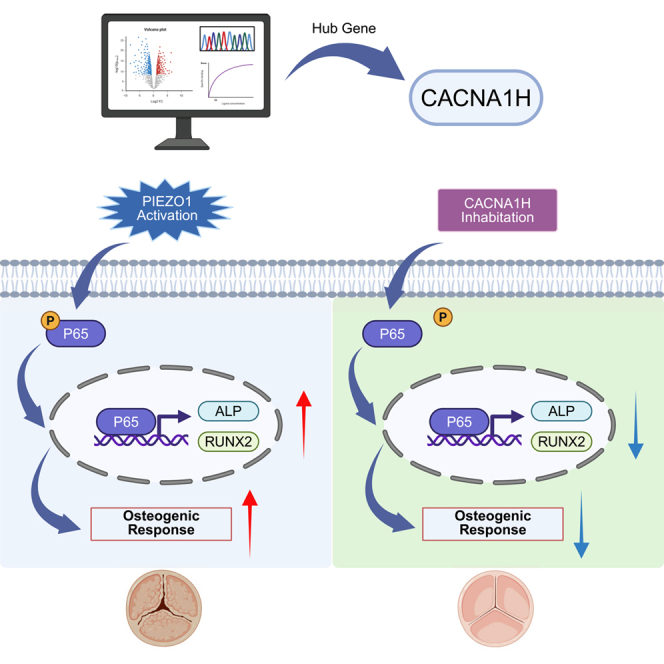

## Introduction

Calcific aortic valve disease (CAVD) is a cardiovascular disorder characterized by fibrosis or calcium deposition in the valve leaflets, with high incidence and mortality rates.^1^^,^^2^ Aortic valve interstitial cells (AVICs) are integral to the pathogenesis of CAVD. Exposure to pro-inflammatory cytokines or altered mechanical stress induces a phenotypic transition of these cells from a quiescent state to a myofibroblast-like or osteoblast-like phenotype. This transition is characterized by the upregulation of key proliferative and osteogenic markers, including alkaline phosphatase (ALP) and runt-related transcription factor 2 (RUNX2), which are indicative of cellular differentiation and active remodeling processes.^3^^,^^4^ Consequently, exploring strategies to inhibit these molecular pathways is essential for the development of innovative therapeutic approaches.

The responsiveness of cardiac valve cells to mechanical stimuli plays a crucial role in maintaining the structural integrity and functional competence of valve leaflets. Physiological mechanical signals are essential for normal organ development and repair, whereas persistent pathological mechanical forces can induce detrimental changes. Mechanical injury to the aortic valve can trigger myofibroblastic or osteoblastic differentiation in AVICs, leading to the deposition of extracellular matrix components and disruption of cellular homeostasis. During tissue remodeling, immune cells are recruited and release inflammatory mediators, such as IL-4 and TGF-β, which exacerbate the inflammatory response.^5^^,^^6^ These mediators, in turn, activate the NF-κB signaling pathway through p65 phosphorylation, thereby amplifying osteogenic processes in the aortic valve.^7^^,^^8^ Consequently, targeting the NF-κB signaling pathway may offer a promising therapeutic strategy for mitigating the progression of CAVD.

**Figure 1:**
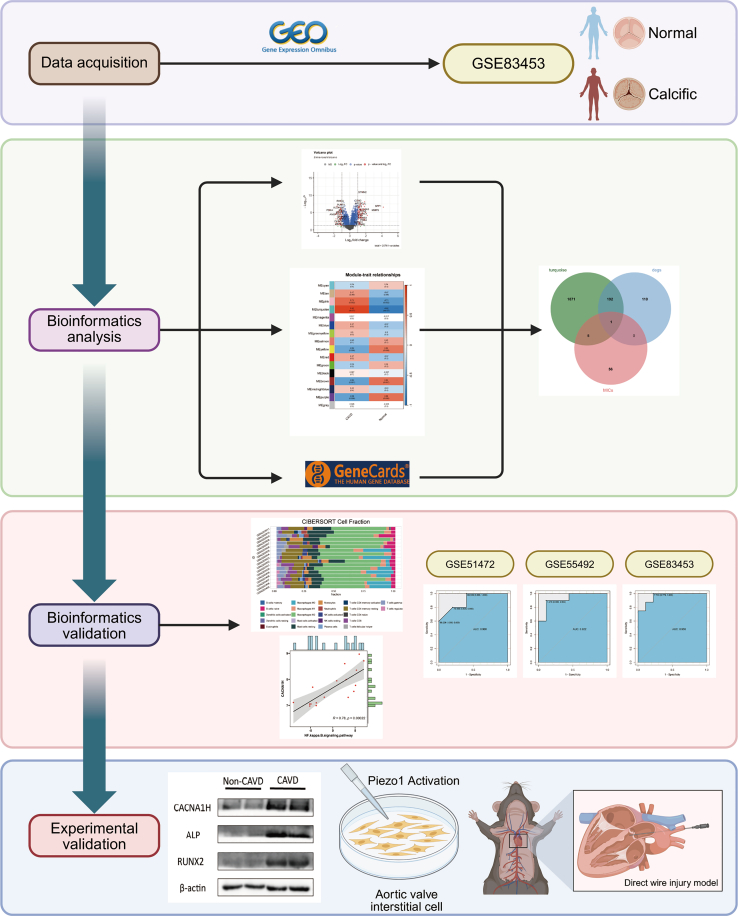
Schematic flow chart of the study design

Cells transduce mechanical stimuli into electrical or chemical signals through mechanically activated ion channels, which are capable of sensing mechanical forces such as tension, compression, and fluid shear stress.^5^ In eukaryotic cells, the PIEZO family of proteins, along with TREK-1/2, TRAAK, and OSCA/TMEM63, function as primary mechanotransducers, while other mechanosensitive ion channels (MICs) serve as downstream effectors of these primary transducers.^9^^,^^10^ Previous studies, including our own, have demonstrated that PIEZO1 plays a pivotal role in modulating glutamine metabolism in AVICs, thereby promoting the onset and progression of CAVD.^11^ Furthermore, it has been suggested that TRPV4 regulates the TGF-β signaling pathway, thereby contributing to CAVD development.^12^^,^^13^ However, the potential involvement of other MICs and their related genes in the pathogenesis of CAVD remains poorly understood. This study aimed to identify a marker gene associated with MIC-related genes (MICRGs) in CAVD through gene expression profiling and bioinformatics approaches and further explored its potential role in the pathogenesis of CAVD. The flowchart of our research is shown in Figure 1.

## Results

### Identification of DEGs and functional enrichment analysis

**Figure 2:**
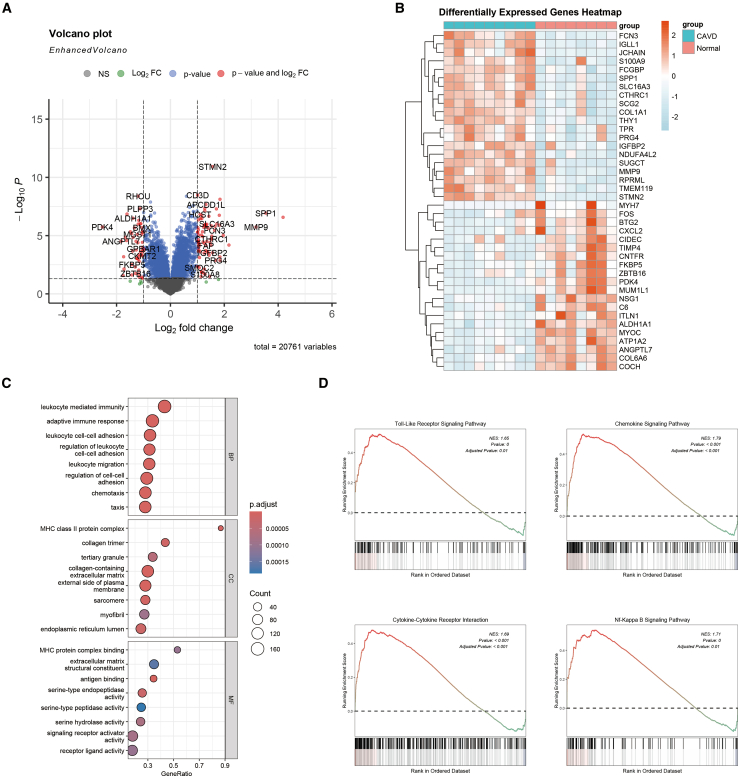
Differentially expressed gene analysis (A) Volcano plot illustrating the differentially expressed genes identified from the GSE83453 dataset, with adjusted *p*-values and |log2 fold change (FC)| > 1. (B) Heatmap depicting the 20 most significantly differentially expressed genes. (C) GO enrichment analysis represented as a bubble plot. (D) KEGG pathway enrichment analysis.

A total of 303 differentially expressed genes (DEGs) were identified in the GSE83453 training dataset, and their expression patterns were visualized using volcano plots and heatmaps (Figures 2A and 2B). Subsequently, Gene Ontology (GO) and Kyoto Encyclopedia of Genes and Genomes (KEGG) pathway enrichment analyses were performed on the DEGs. In the biological process (BP) analysis, the DEGs were predominantly enriched in pathways related to leukocyte-mediated immunity, the adaptive immune response, and leukocyte cell-cell adhesion. The cellular component (CC) analysis revealed enrichment in structures such as the MHC class II protein complex, collagen trimer, and tertiary granules. In the molecular function (MF) analysis, the DEGs were enriched in functional components, including MHC protein complex binding and structural constituents of the extracellular matrix (Figure 2C). Additionally, KEGG pathway analysis indicated significant enrichment of DEGs in key signaling pathways, including the Toll-like receptor (TLR) signaling pathway and the NF-κB signaling pathway (Figure 2D).

### Hub genes screening

**Figure 3:**
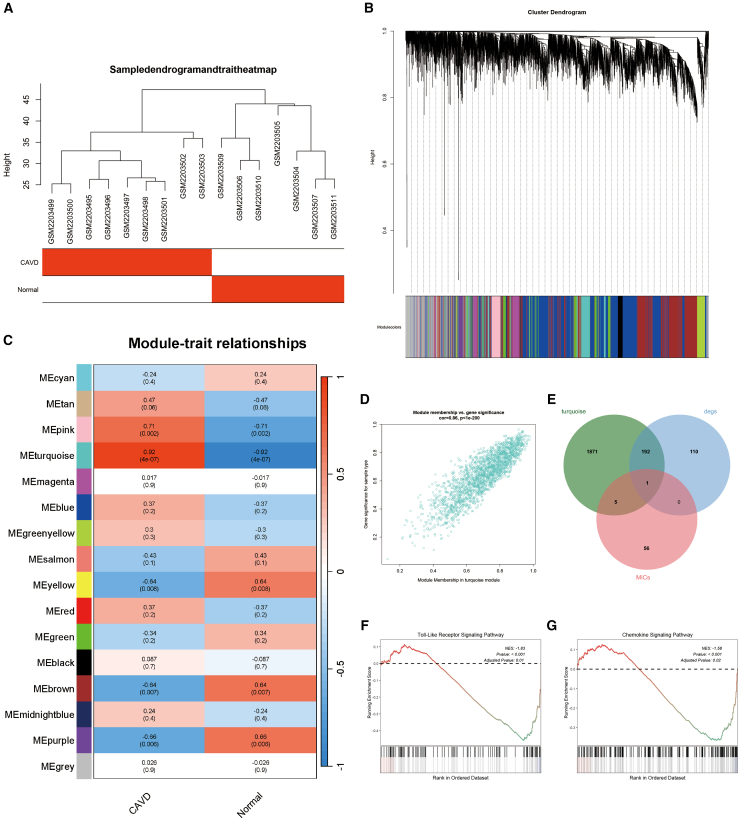
Key gene identification and analysis (A) Hierarchical clustering based on gene expression levels from the GSE83453 dataset. (B) Visualization of original and merged gene modules beneath the clustering tree derived from GSE83453. (C) Heatmap illustrating the correlation between module-characteristic genes and the occurrence of CAVD. (D) Scatterplot depicting the correlation between the turquoise module and gene significance. (E) Venn diagram displaying the overlap among the turquoise module, DEGs, and MICRGs. (F) GSEA of the Toll-like receptor signaling pathway. (G) GSEA of the chemokine signaling pathway.

To further investigate the key genes involved in CAVD, WGCNA was employed to identify modules associated with the disease. After excluding one outlier sample, the sample clustering and gene clustering diagrams for nine patients with CAVD and seven normal controls are presented in Figures 3A and 3B. Figure 3C illustrates the correlation between CAVD and gene modules, demonstrating a robust association between the turquoise module and CAVD (r = 0.92, *p* < 0.05). Furthermore, a significant correlation was observed between the turquoise module and gene significance (r = 0.82, *p* < 0.05, Figure 3D). Based on these findings, it can be concluded that the turquoise module is strongly associated with CAVD. Additionally, 62 MICRGs were screened from the Genecard database and intersected with DEGs and genes from the turquoise module, identifying *CACNA1H* as a key gene (Figure 3E). KEGG enrichment analysis of *CACNA1H* revealed its involvement in signaling pathways such as the TLR signaling pathway and the chemokine signaling pathway, both of which are implicated in CAVD (Figures 3F and 3G).

### Correlation analysis and validation

**Figure 4:**
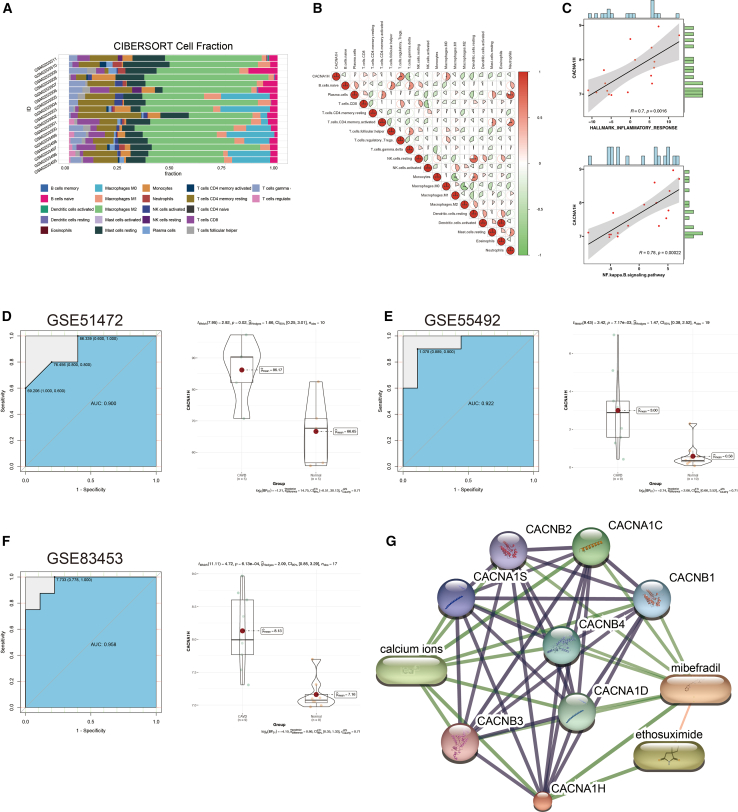
Immune infiltration characteristics and external validation (A) Histogram illustrating the immune cell infiltration profiles in 22 patients with CAVD from the GSE83453 dataset. (B) Heatmap depicting the correlation between *CACNA1H* expression and immune cell infiltration levels. (C) Analysis of the correlation between *CACNA1H* expression and inflammation-related signaling pathways. (D) ROC curve analysis alongside gene expression data of *CACNA1H* in the GSE51472 dataset. (E) ROC curve analysis alongside gene expression data of *CACNA1H* in the GSE55492 dataset. (F) ROC curve analysis alongside gene expression data of *CACNA1H* in the GSE83453 dataset. (G) Protein-protein interaction network analysis of *CACNA1H* and its interacting partners.

To assess immune infiltration, the CIBERSORT algorithm was utilized to evaluate immune cell types among the DEGs, followed by correlation analyses between *CACNA1H* and immune cell populations (Figure 4A). The results revealed that *CACNA1H* exhibited a positive correlation with regulatory T cells (Tregs) and M0 macrophages, while showing a negative correlation with γδ T cells (Figure 4B). Figure 4C illustrate significant positive correlations between *CACNA1H* and the inflammatory response (r = 0.70, *p* < 0.05) as well as the NF-κB signaling pathway (r = 0.78, *p* < 0.05). Validation of these findings was subsequently performed using the GSE51472, GSE55492, and GSE83453 datasets. Receiver operating characteristic (ROC) curve analysis demonstrated that *CACNA1H* effectively distinguished CAVD from normal controls (Figures 4D–4F). Figure 4G presents *CACNA1H*-associated genes and potential therapeutic targets.

### CACNA1H mediates AVIC osteogenic differentiation

**Figure 5:**
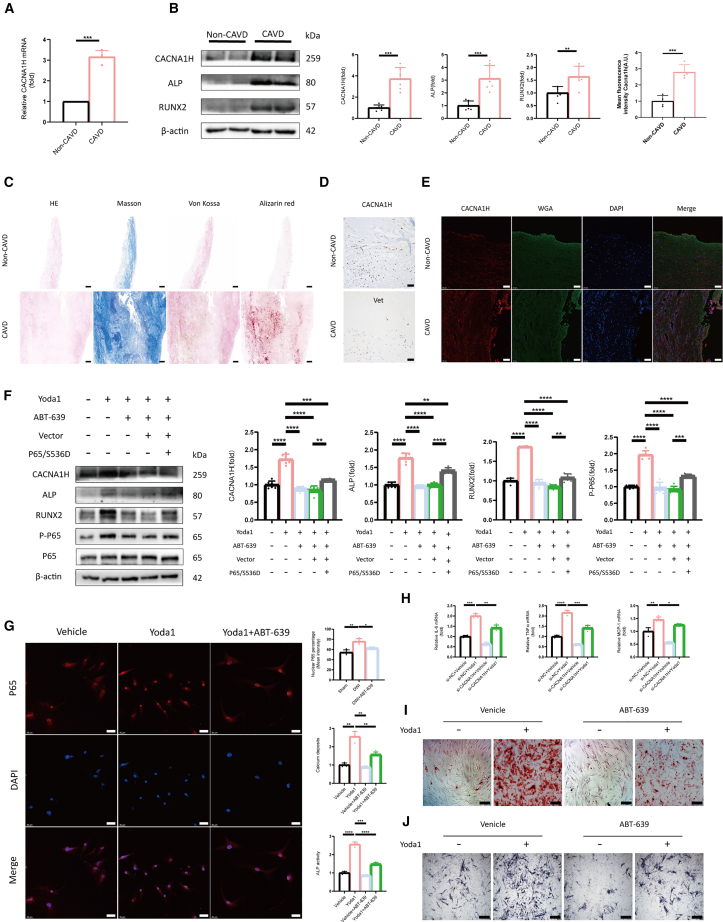
CACNA1H is upregulated in human CAVD and its pharmacological inhibition attenuates calcification via suppression of the NF-κB pathway in AVICs (A) *CACNA1H* mRNA levels in human aortic valves from CAVD and normal controls (*n* = 4). (B) Representative immunoblots and quantitative analysis of CACNA1H protein expression in CAVD and non-CAVD groups (*n* = 6). (C) Histological characterization of human aortic valve leaflets stained with H&E, Masson’s trichrome, Von Kossa, and Alizarin red, demonstrating calcification (Scale bars, 250 μm). (D) Immunohistochemical staining showed CACNA1H protein localization in CAVD and non-CAVD aortic valve tissues (Scale bars, 50 μm). (E) Immunofluorescence staining of CACNA1H in valve tissues (*n* = 4, Scale bars, 50 μm). (F) Representative immunoblots and quantitative analysis showing that ABT-639 downregulates Yoda1-induced expression of osteogenic markers and P65 activation; this effect was reversed by the constitutively active P65 S536D mutant (*n* = 6). (G) Immunofluorescence analysis of NF-κB P65 subcellular localization confirms that ABT-639 impairs its nuclear translocation following Yoda1 stimulation (*n* = 4, Scale bars, 50 μm). (H) Real-time PCR analysis shows that ABT-639 reduces Yoda1-induced mRNA expression of inflammatory cytokines (IL-6, TNF-α, MCP-1) (*n* = 4). (I) Alizarin red staining reveals that ABT-639 significantly reduces Yoda1-induced mineralization nodule formation in VICs (*n* = 4, Scale bars, 200 μm). (J) ALP staining indicated that ABT-639 markedly inhibited Yoda1-induced ALP activity in normal VICs (*n* = 4, Scale bars, 500 μm). Data are presented as means ± SEM. Statistical significance was determined using Student’s *t* test or one-way ANOVA. ∗*p* < 0.05, ∗∗*p* < 0.001, ∗∗∗*p* < 0.001, ∗∗∗∗*p* < 0.0001.

To further explore the role of *CACNA1H* in human aortic valve pathology, both normal and calcified aortic valve samples were obtained. Quantitative PCR of extracted valve tissue revealed significantly elevated mRNA levels of *CACNA1H* in calcified valves (Figure 5A). Western blot analysis demonstrated increased expression of CACNA1H, as well as the osteogenic markers ALP and RUNX2, in calcified tissues (Figure 5B). HE and Masson staining revealed significant thickening of the calcified aortic valves, while Von Kossa and alizarin red staining confirmed the deposition of calcium salts (Figure 5C). Immunohistochemical analyses revealed that CACNA1H-positive cells are predominantly localized within the ventricular layer of the aortic valve (Figure 5D). Immunofluorescence analysis indicated an upregulation of CACNA1H expression in calcified tissue (Figure 5E).

The optimal concentration of ABT-639 was identified using a series of concentration gradients, with 10 μM selected for subsequent experiments (Figures S1A–S1C). To investigate whether CACNA1H plays a role in the osteogenic differentiation of AVICs, cells were treated with Yoda1, a specific agonist of the MIC PIEZO1 (Figures 5F and S2A). Silencing of *CACNA1H* (si*CACNA1H*) also markedly attenuated the osteogenic phenotype induced by PIEZO1 (Figure S2B). Western blot analysis revealed that Yoda1 treatment led to a significant increase in CACNA1H protein levels. Moreover, Yoda1 stimulation was associated with enhanced phosphorylation of P65, as well as upregulation of osteogenic markers, ALP and RUNX2. Co-treatment with ABT-639, a CaV3.2 channel blocker, partially attenuated Yoda1-induced phosphorylation of p65 and the expression of osteogenic markers in AVICs. The sustained activation of P65 phosphorylation, induced via the P65/s536d plasmid, effectively prevents the downregulation of ALP and RUNX2 by ABT-639. The results presented in Figure 5H demonstrate that stimulation with Yoda1 upregulates the transcriptional expression of inflammatory cytokines, including IL-6, TNF-α, and monocyte chemoattractant protein-1 (MCP-1), in AVICs. Conversely, si*CACNA1H* markedly attenuates the expression levels of these cytokines. Alizarin red staining results indicate that ABT-639 significantly reduces Yoda1-induced calcium salt deposition (Figure 5I). Furthermore, ALP staining demonstrates that ABT-639 decreases Yoda1-induced ALP activity (Figure 5J). The results suggest that the inhibition of CACNA1H mitigates osteogenic differentiation in AVICs by attenuating P65 phosphorylation.

### CACNA1H promotes aortic valve calcification *in vivo*

**Figure 6:**
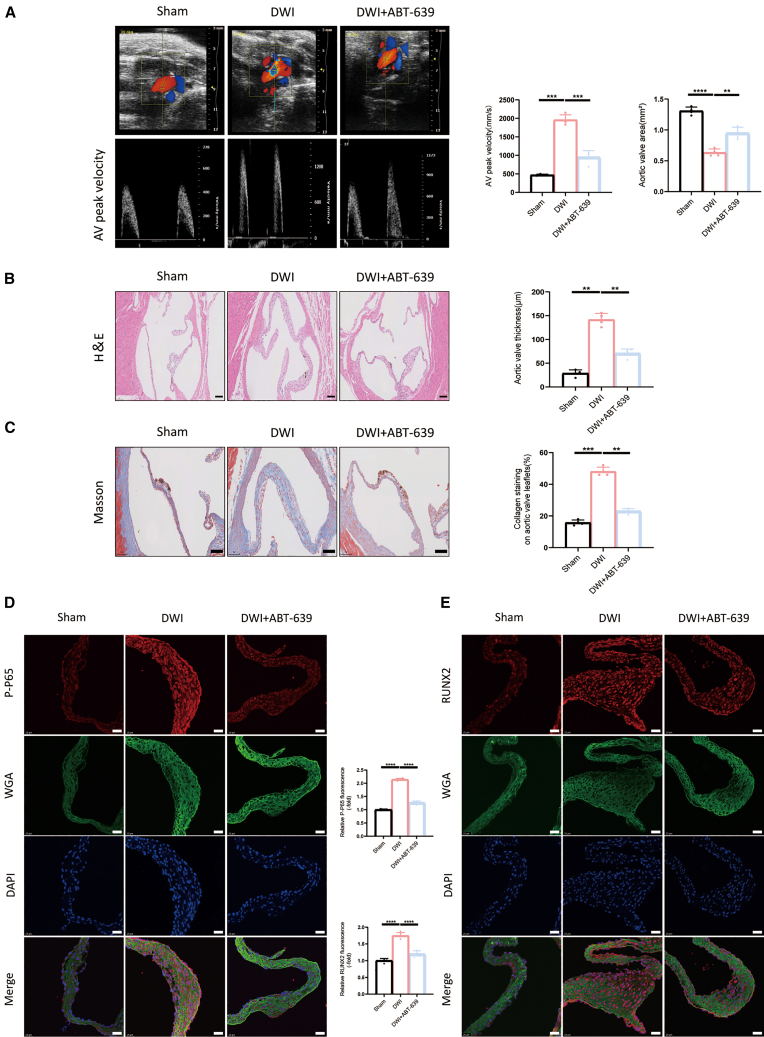
Inhibition of CACNA1H prevents DWI-induced aortic valve calcification *in vivo* (A) Aortic valve peak velocity and aortic valve area in C57BL/6J mice treated as Sham, DWI + vehicle, or DWI + ABT-639 treatment (*n* = 4). (B) H&E staining of aortic valves following wire injury (*n* = 4, Scale bars, 100 μm). (C) Masson’s trichrome staining of aortic valves (*n* = 4, Scale bars, 100 μm). (D and E) Representative immunofluorescence images of P-P65 and RUNX2 in aortic valves from DWI-treated mice (*n* = 4, Scale bars, 25 μm). Data are presented as means ± SEM. Statistical significance was determined using one-way ANOVA. ∗*p* < 0.05, ∗∗*p* < 0.001, ∗∗∗*p* < 0.001, ∗∗∗∗*p* < 0.0001.

To elucidate the role of CACNA1H in aortic valve calcification, an aortic valve injury model was established in direct wire injury (DWI) mice, followed by alternate-day intraperitoneal administration of either vehicle or ABT-639. After a 12-week period, echocardiographic assessment was conducted. The results demonstrated that, compared to the sham-operated group, DWI mice exhibited significantly elevated peak transvalvular blood flow velocities and reduced aortic valve area (Figure 6A). Treatment with ABT-639 significantly attenuated these alterations.

Histological analysis revealed pronounced thickening of the aortic valve in DWI mice, a phenomenon that was mitigated by ABT-639 treatment (Figure 6B). Additionally, Masson staining indicated that ABT-639 reduced collagen deposition in the aortic valve induced by DWI (Figure 6C). Immunofluorescence analysis further demonstrated that ABT-639 decreased the expression levels of CACNA1H, P-P65, as well as osteogenic markers in the aortic valves of the mice (Figures 6D, 6E, and S3A). Alizarin red staining results indicate that inhibition of CACNA1H reduces calcium salt deposition in the aortic valve of mice (Figure S4A). The above results indicate that inhibition of CACNA1H can attenuate the aortic valve calcification process.

## Discussion

CAVD is a progressive condition characterized by aortic valve calcification, leading to valve stenosis and heart failure. Despite its high prevalence in the elderly population and its association with poor prognosis, the mechanisms driving CAVD are still not fully understood, and there are limited treatment options available.^14^ Previous studies have primarily focused on the mechanical stress and inflammatory pathways involved in CAVD pathogenesis.^1^ In this context, our study presents *CACNA1H* as a critical biomarker and potential therapeutic target, shedding light on its crucial role in immune activation and osteogenic differentiation during CAVD progression.

This study identified *CACNA1H* as a candidate biomarker and potential therapeutic target through the analysis of transcriptomic data from the GEO database. *CACNA1H* is significantly upregulated in pathological aortic valve tissue, where it appears to be involved in immune activation and inflammatory responses. Furthermore, the data reveal a strong correlation between *CACNA1H* expression and the infiltration of various immune cell types, notably Treg cells, gamma delta T cells, and M0 macrophages. Treg cells, known for their immunosuppressive functions, play a pivotal role in maintaining immune tolerance. The observed increase in Treg cells suggests that *CACNA1H* might facilitate immune homeostasis in the valve microenvironment by preventing excessive immune activation.^15^ Interestingly, the reduction in gamma delta T cells could indicate a shift toward a more suppressive immune profile, potentially mediated by *CACNA1H* regulation. M0 macrophages, as precursor cells to pro-inflammatory M1 macrophages, might also be influenced by *CACNA1H* in modulating the inflammatory landscape in CAVD.^16^ These findings are consistent with previous studies suggesting that immune cell infiltration is a key driver of valve calcification and that immune modulation could offer relevant therapeutic avenues for CAVD.

The expression of CACNA1H was significantly upregulated in both experimental DWI model mice and diseased aortic valve tissue. Inhibition of CACNA1H notably attenuated the osteogenic response in AVICs and alleviated valve thickening in DWI mice. Mechanistically, suppression of CACNA1H mitigated osteogenesis by reducing the phosphorylation of P65. These findings provide valuable insights into the molecular mechanisms underlying the progression of CAVD, particularly in the context of mechanical sensitivity.

*CACNA1H* is a member of the T-type voltage-gated calcium channel family, encoding the α-subunit Cav3.2.^17^ Its expression in cardiac tissue is highest during embryonic development and subsequently declines significantly after the neonatal period. Overexpression of *CACNA1H* leads to intracellular calcium accumulation, which disrupts calcium signaling and contributes to cellular dysfunction.^18^^,^^19^ Previous studies have demonstrated that mechanically induced stress, pressure overload, or angiotensin II infusion result in elevated CACNA1H expression, which plays a critical role in the development of pathological myocardial hypertrophy and arrhythmogenic alterations.^20^^,^^21^ Furthermore, inhibition of CACNA1H has been shown to protect the heart by reducing myocardial cell apoptosis, likely through the downregulation of endoplasmic reticulum stress-related pathways, including PERK and ATF4.^22^ Despite extensive research on the role of CACNA1H in cardiac diseases, its involvement in CAVD remains unexplored.

Mechanical stress plays a crucial role in the development and progression of CAVD. Our previous research has demonstrated that Yoda1, a selective agonist of PIEZO1, promotes osteogenic responses through histone acetylation mediated by Yap-dependent glutamine metabolism.^11^ Consequently, Yoda1 serves as an exogenous stimulus that mimics mechanical stress *in vitro*. Notably, our findings reveal that activation of PIEZO1 induces the upregulation of *CACNA1H* gene expression. This suggests that calcium influx via PIEZO1 may interact with T-type calcium channels through calcium signaling pathways, thereby modulating CACNA1H channel activity and enhancing its protein expression. Furthermore, PIEZO1 and CACNA1H may exhibit synergistic interactions within specific intracellular compartments, where alterations in membrane potential could potentially influence CACNA1H expression levels.

P65 is a critical component of the NF-κB signaling pathway, serving as a key transcription factor in cellular responses to a variety of physiological and pathological stimuli. Under basal conditions, NF-κB exists as an inactive dimer complexed with the inhibitory protein IκB, which confines it to the cytoplasm. Following activation by external signals, IκB undergoes phosphorylation and subsequent degradation, enabling the translocation of NF-κB dimers, such as P65/P50, into the nucleus. Once in the nucleus, these dimers initiate the transcription of various target genes involved in immune responses and cell survival.^23^

Recent studies have demonstrated that inhibition of P65 phosphorylation can slow the progression of CAVD, highlighting the significance of this pathway in disease pathogenesis.^2^^,^^7^ Upon activation, P65 drives the transdifferentiation of AVICs into osteoblast-like cells, thereby enhancing the cellular potential for calcification. This effect is primarily mediated by the upregulation of calcification-related proteins, such as osteocalcin, a well-established marker of osteogenesis.^24^ Moreover, P65 phosphorylation plays a pivotal role in modulating the inflammatory response within the valve microenvironment. It alters the secretion of pro-inflammatory cytokines and contributes to extracellular matrix remodeling, both of which are integral to the development of calcific nodules. Consequently, the activation of P65 not only promotes osteogenic differentiation but also exacerbates the pathological conditions conducive to calcium salt deposition within the valve.

Our data demonstrate that inhibition of CACNA1H attenuates Yoda1-induced osteogenic responses by reducing p65 phosphorylation. Previous studies have shown that aberrant activation of CACNA1H leads to increased calcium influx, which activates the NF-κB/P65 pathway, resulting in the upregulation of inflammatory factors such as IL-6 and TNF-α, consistent with our findings.^25^^,^^26^ These observations suggest that CACNA1H plays a critical role in regulating the NF-κB signaling pathway and influencing the inflammatory response.

In conclusion, the results of our study suggest that *CACNA1H* plays a pivotal role as a pathogenic factor in the development of CAVD. Specifically, CACNA1H facilitates osteogenic processes by activating the NF-κB signaling pathway, primarily through phosphorylation of the P65 subunit. Furthermore, mechanical stress-induced aortic valve calcification can be effectively mitigated through the use of selective inhibitors targeting CACNA1H. These findings underscore the potential for candidate therapeutic interventions in the management of CAVD.

### Limitations of the study

Our study has several limitations that warrant consideration. First, we focused primarily on the function of CACNA1H in valvular interstitial cells (VICs) and did not investigate its potential role in valvular endothelial cells (VECs), which are critical regulators of valve homeostasis. Future studies should explore the crosstalk between CACNA1H-mediated signaling in VECs and VICs to fully understand the complex cellular interactions in CAVD. Second, while we demonstrated that CACNA1H inhibition suppresses NF-κB activation, the precise molecular mechanism linking altered calcium homeostasis to p65 phosphorylation remains to be elucidated. Investigating the involvement of calcium-sensitive kinases (e.g., CaMKII or PKC) would provide further mechanistic insight into this signaling cascade. Third, currently available human genetic evidence supporting a direct role of *CACNA1H* in this disease remains limited, which should be addressed in future well-powered genetic and clinical studies.

## Resource availability

### Lead contact

Requests for further information and resources should be directed to and will be fulfilled by the lead contact, Qingchun Zeng (qingchunzeng@smu.edu.cn).

### Materials availability

This study did not generate new unique reagents.

### Data and code availability

• The transcriptomic data used in this study were obtained from the GEO database (https://www.ncbi.nlm.nih.gov/geo/).
• All original code has been deposited on GitHub and is publicly available as of the date of publication. The repository is listed in the key resources table.
• Any additional information required to reanalyze the data reported in this paper is available from the lead contact upon request.

## Acknowledgments

This work was supported by the 10.13039/100014718National Natural Science Foundation of China (82070403, 82270374, U25D9020), the 10.13039/501100021171Guangdong Basic and Applied Basic Research Foundation (2021A0505030031, 2024A1515011387), and the 10.13039/501100014945Guangzhou Science and Technology Plan Project (2023B01J1011, 2023B03J1243).

## Author contributions

J.C.: conceptualization, formal analysis, visualization, writing – original draft, writing – review and editing. L.W.: investigation, methodology visualization. W.L.: resources, investigation. S.L.: methodology, visualization. X.D.: project administration, formal analysis, methodology. S.J.: investigation, resources. Z.Z.: data curation, resources. Q.Z.: conceptualization, funding acquisition, supervision, writing – review and editing.

## Declaration of interests

The authors declare that they have no competing interests.

## STAR★Methods

### Key resources table

**Table:** 

REAGENT or RESOURCE	SOURCE	IDENTIFIER
**Antibodies**		

CACNA1H	HUABIO	Cat# ER65238; RRID: AB_3750174
ALP	Abclonal	Cat# A0514; RRID: AB_2861462
RUNX2	HUABIO	Cat# ET1612-47; RRID: AB_2924311
P65	Abclonal	Cat# A19653; RRID: AB_2862717
P-P65	Cell Signaling	Cat# 3033; RRID: AB_331284
β-actin	Proteintech	Cat# 66009-1; RRID: AB_2687938

**Chemicals, peptides, and recombinant proteins**		

Collagenase II	Sigma-Aldrich	Cat# 1148090
ABT-639	MedChemExpress	Cat# HY-19721
Yoda1	MedChemExpress	Cat# HY-18723
FITC Conjugate WGA	Beyotime	Cat# Y262488
Alizarin red S	Leagene	Cat# DS0058

**Critical commercial assays**		

RNA isolater Total RNA Extraction Reagent	Vazyme	Cat# R401-01
ChamQ Blue Universal SYBR qPCR Master Mix	Vazyme	Cat# Q312-02
HiScript II Q RT SuperMix for qPCR	Vazyme	Cat# R222-01
Masson’s Trichrome Stain Kit	Solarbio	Cat# G1340
Calcium Stain Kit(Von Kossa Method)	Solarbio	Cat# G3282
BCIP/NBT Alkaline Phosphatase Color Development Kit	Beyotime	Cat# C3206
Cell Counting Kit-8	Beyotime	Cat# C0039

**Deposited data**		

RNA-seq data	GEO	GSE83453
RNA-seq data	GEO	GSE51472
RNA-seq data	GEO	GSE55492

**Experimental models: Cell lines**		

Aortic valve interstitial cells	Nanfang Hospital	N/A

**Experimental models: Organisms/strains**		

C57BL/6J mice	VitalRiver	N/A

**Oligonucleotides**		

CACNA1H sense (5′-CGCCACCTTCAGCAACTTCGGCAT-3′)	Tsingke	https://doi.org/10.1182/blood-2003-07-2482
CACNA1H antisense (5′-ATCTCCACCTCCTGCAGCGG-3′)	Tsingke	https://doi.org/10.1182/blood-2003-07-2482

**Recombinant DNA**		

P65/S536D	Geneyuan	Custom construct

**Software and algorithms**		

Custom analysis code	This paper; GitHub	https://github.com/WayttC/Jun.git
GraphPad Prism 10	GraphPad	N/A
ImageJ	NIH	N/A

### Experimental model and study participant details

#### Animal models

All animal experiments were approved by the Ethics Committee of Nanfang Hospital (IACUC-LAC-20220705-002). The animals were housed under specific pathogen-free (SPF) conditions in the animal facilities of Nanfang Hospital, with unrestricted access to food and water. To establish a CAVD model, 12-week-old C57BL/6 male mice were subjected to a guidewire-induced injury model. The procedure involved anesthetizing the mice, performing a carotid artery dissection, and advancing a spring steel wire into the ascending aorta. The wire was then used to repeatedly scrape the aortic valve 50 times and rotate 100 times within the valvular ventricle to induce localized injury to the valve. After the wire was removed, the carotid artery was ligated. The control group underwent the same procedure, with the exception of the valve injury.

#### Primary cell cultures

This study received approval from the Human Ethics Committee of Nanfang Hospital and fully complied with the ethical guidelines for human tissue use as stipulated by the Declaration of Helsinki (NFEC2021064). Prior to surgery, all participants provided written informed consent. Calcified aortic valve tissue samples were collected from patients undergoing aortic valve replacement, while normal aortic valve tissue samples were sourced from the discarded hearts of patients undergoing heart transplantation. The baseline characteristics of the donors are detailed in Table S1. AVICs were isolated from human aortic valve tissues, washed with phosphate-buffered saline (PBS), and subsequently incubated in a culture medium containing collagenase (type II, 2.5 mg/mL; Sigma-Aldrich, 1148090, USA) for enzymatic digestion at 37°C in a 5% CO_2_ atmosphere for 12 h. Following digestion, the supernatant was discarded by centrifugation at 1000 rpm for 5 min. The remaining tissue was resuspended in high-glucose Dulbecco’s Modified Eagle Medium (DMEM), supplemented with 5% fetal bovine serum (FBS; Gibco, Invitrogen, Carlsbad, CA, USA), and cultured at 37°C in a 5% CO_2_ incubator. Cell passages were maintained for 3 to 5 generations prior to use in subsequent experiments. A concentration of 5 μM Yoda1 (MedChemExpress, HY-18723, USA) was chosen to stimulate AVICs *in vitro*, thereby simulating mechanical stress to induce calcification of AVICs.^11^ The P65/S536D plasmid was obtained from Geneyuan Biotechnology.

### Method details

#### Data collection

Sequence data pertaining to aortic valve tissue from both normal and CAVD subjects were retrieved from the Gene Expression Omnibus (GEO) database (https://www.ncbi.nlm.nih.gov/geo/), specifically from the datasets GSE83453, GSE51472, and GSE55492. The GSE83453 dataset includes data from nine CAVD patients and eight healthy controls, serving as the primary test dataset. In contrast, the GSE51472 and GSE55492 datasets were employed for subsequent validation purposes.

#### Differential gene expression and functional annotation

Differential gene expression analysis was performed using the “limma” package in R to identify differentially expressed genes (DEGs) between the CAVD group and healthy controls. Statistical significance was determined based on adjusted *p*-values and a |log_2_ fold change (FC)|> 1 threshold. For the visualization of DEGs, the “ggplot2” and “pheatmap” packages were employed. Furthermore, the “clusterProfiler” package was utilized to conduct Kyoto Encyclopedia of Genes and Genomes (KEGG) and Gene Ontology (GO) enrichment analyses of the identified DEGs. The “org.Hs.e.g.,.db” gene set database was used as the reference for these analyses. An adjusted *p*-value of <0.05 was considered indicative of significantly enriched pathways or terms. The figures illustrating the GO and KEGG results were generated using the “ggplot2” and “GseaVis” package.

#### Weighted gene co-expression network analysis (WGCNA)

To identify gene modules with high correlation and screen for potential biomarker genes, the WGCNA was conducted on the training set data using the “WGCNA” package, following a structured workflow. Initially, outlier genes and samples were excluded to optimize the quality of the data. Subsequently, a scale-free co-expression network was constructed by transforming the correlation matrix into an adjacency matrix, achieved through the selection of an optimal soft threshold. Based on this adjacency matrix, a Topological Overlap Matrix (TOM) was generated to facilitate the identification of gene modules.

Upon obtaining the gene modules, the corresponding module eigengenes (MEs) were extracted, which represent the first principal component of each module’s expression profile. To assess the relationship between the identified modules and clinical features, module-trait correlations were performed, revealing modules with the most significant positive or negative associations. The biological relevance of each module was then determined by evaluating module membership (MM) and gene significance (GS) within the respective modules.

#### Immune infiltration analysis and protein-protein interaction (PPI) networks

The CIBERSORT algorithm was utilized to assess the differences in immune cell infiltration between patients with CAVD and healthy controls. Additionally, a gene-immune association analysis was conducted using the “corplot” package to explore potential correlations. The STRING database is employed in constructing protein interaction networks.

#### RNA extraction and quantitative real-time PCR (qRT-PCR) assay

Total cellular RNA was extracted using the RNA Isolator Total RNA Extraction Reagent (Vazyme, R401-01, China), with 1 mL of reagent per 1 × 10^7^ to 3 × 10^7^ cells. Following the addition of 200 μL of chloroform, the mixture was thoroughly vortexed and incubated at 4°C for 5 min. The solution was then subjected to centrifugation at 11,200 rpm for 15 min. The resulting supernatant was carefully removed, and an equal volume of isopropanol was added to the pellet, followed by incubation at 4°C for 10 min. A second centrifugation was performed at 11,200 rpm for 10 min. The RNA pellet was subsequently washed with 75% ethanol and centrifuged again at 11,200 rpm for 5 min. After discarding the supernatant, the RNA was resuspended in RNA-free water.

Following RNA extraction, reverse transcription was carried out using the HiScript II Q RT SuperMix (Vazyme, R222, China) to synthesize complementary DNA (cDNA) from mRNA. qRT-PCR analysis was performed using the SYBR Green qPCR Master Mix (Vazyme, R312, China). PCR amplification was conducted under standard cycling conditions on the LightCycler 480 System (Roche). Relative mRNA expression levels were normalized to the reference gene β-actin, and gene expression was quantified using the 2ˆ(-ΔΔCt) method.

#### Protein extraction and Western Blot analysis

Protein extraction was conducted at 4°C. For each 100 mg of aortic valve tissue or 1 × 10^7^ cells, 1 mL of RIPA protein lysis buffer, supplemented with protease and phosphatase inhibitors (FUDE Biological Technology, FD1001, FD1002, China), was added. The protein concentration was determined using the BCA Protein Assay Kit (FUDE Biological Technology, FD2001, China), following the manufacturer’s instructions.

Proteins were then separated by 10% SDS-PAGE and transferred to a polyvinylidene fluoride (PVDF) membrane (Millipore, ISEQ00010, USA). Following transfer, the membrane was blocked with 5% skimmed milk powder for 1 h at room temperature. Subsequently, the membrane was incubated overnight at 4°C with the primary antibody. The next day, after washing the membrane, it was incubated with horseradish peroxidase-conjugated secondary antibody (FUDE Biological Technology, FDR007, FDM007, China) at room temperature for 1 h. Protein bands were visualized using the FDbio-Dura ECL Kit (FUDE Biological Technology, FD8020, China) and quantified using ImageJ software.

#### Alizarin red staining

Upon achieving the appropriate cell density, AVICs were cultured in osteogenic growth medium and indicated interventions, supplemented with β-glycerophosphate (10 mM), dexamethasone (10 nM), cholecalciferol (4 μg/mL), and calcium chloride (8 mM) for a period of 14 days. The medium was refreshed every three days during the stimulation period. Following three washes with phosphate-buffered saline (PBS), the cells were fixed using 4% paraformaldehyde for 15 min and subsequently stained with Alizarin Red S solution (Leagene, DS0058, China) for 30 min to assess mineralization. Imaging was conducted using an Olympus CKX41 microscope (Olympus, Japan). For quantitative analysis, the stained cells were eluted with 10% acetic acid at 75°C and the absorbance was measured spectrophotometrically at a wavelength of 450 nm.

#### ALP staining

Upon reaching confluence, the cells were cultured in osteogenic growth medium and indicated interventions for 7 days, with the medium being replaced every three days to maintain optimal conditions for differentiation. Subsequently, the cells were washed three times with PBS and fixed with 4% paraformaldehyde for 15 min to preserve cellular morphology. ALP activity was assessed by performing staining for 2 h using the BCIP/NBT alkaline phosphatase color development kit (Beyotime, C3206, China), which allowed for the visualization of enzyme activity. Imaging of stained cells was conducted using an Olympus CKX41 microscope (Olympus, Japan).

#### Immunofluorescence (IF) staining

Aortic valve tissue, obtained from the recipient and embedded in paraffin, was sectioned into 5-μm thick slices. To prevent non-specific binding, the sections were first blocked with 5% bovine serum albumin (BSA) for 1 h. The slides were then incubated overnight at 4°C with an anti-CACNA1H primary antibody. On the following day, after thorough washing with PBS, the sections were incubated for 1 h at room temperature with a fluorescently labeled secondary antibody. After additional washing, the slides were stained with DAPI for 30 min to counterstain the nuclei. Following the final wash, the slides were mounted and visualized using a Leica SP8 (Germany) confocal microscope.

#### Hematoxylin and eosin (H&E) staining

Following paraffin embedding and dehydration, aortic valve sections were first washed with PBS to remove excess embedding medium. The sections were then stained with hematoxylin for 3 min, followed by a wash with PBS, and subsequently stained with eosin for 1 min. The stained sections were dehydrated using an ethanol gradient and cleared in xylene for 2 min. Finally, images of the stained sections were captured using an Olympus CKX41 microscope (Olympus, Japan).

#### Von Kossa staining

Aortic valve sections were incubated in a 5% silver nitrate solution for 30 min. Subsequently, the sections were exposed to ultraviolet (UV) light for 1 h, washed thoroughly, and treated with a 5% sodium sulfate solution for 2 min. Following counterstaining with neutral red for 3 min, calcium deposition in the aortic valve leaflets was examined using an Olympus CKX41 microscope (Olympus, Japan).

#### Masson’s trichrome staining

Aortic valve sections were prepared for the assessment of collagen deposition. Masson’s trichrome staining was performed using the Masson Trichrome Staining Kit (Servicebio, G1006, China) according to the manufacturer’s protocol. Following staining, the sections were mounted using neutral-balsam resin and subsequently imaged with an Olympus CKX41 microscope (Olympus, Japan).

### Quantification and statistical analysis

All statistical analyses were conducted using GraphPad Prism (version 10.0). Each experiment was performed at least triplicate to ensure reproducibility. Data are expressed as the mean ± Standard error of mean (SEM). Parametric tests, such as t-tests, were employed for data that followed a normal distribution, while non-parametric tests (e.g., Wilcoxon signed-rank test) were applied to data that did not meet the assumption of normality. A *p*-value of less than 0.05 (*p* < 0.05) was considered statistically significant. In all cases a *p* value ≤0.05 was considered to be significant. ∗*p* < 0.05, ∗∗*p* < 0.001, ∗∗∗*p* < 0.001, ∗∗∗∗*p* < 0.0001.
