# Supplementary material for: Exploring CACNA1H as a potential candidate biomarker for calcific aortic valve disease
Source: iScience. 2026 Apr 27;29(5):115616. doi: 10.1016/j.isci.2026.115616 (PMC13137072; doi:10.1016/j.isci.2026.115616)

iScience, Volume *29*

Supplemental information

Exploring CACNA1H as a potential candidate biomarker for calcific aortic valve disease

Jun Chen, Liusheng Wang, Wenyan Li, Shunyi Li, Xiaolin Duan, Sijie Jiang, Zhen Zhang, and Qingchun Zeng

Supplementary Figures

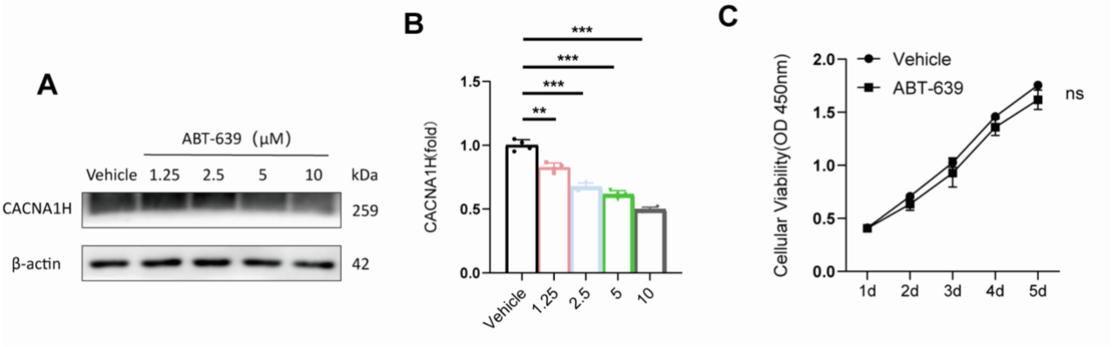

Figure S1: Cytotoxicity of ABT-639 and its effect on CACNA1H protein expression in VICs. (A, B) Human aortic VICs were treated with increasing concentrations of ABT-639 (vehicle, 1.25, 2.5, 5, and 10 μM) for 72 hours. Representative immunoblots and quantitative analysis show CACNA1H protein levels (n = 4). (C) Cell viability assessed by CCK-8 assay in VICs treated with ABT-639 (n = 4). Data are presented as means ± SEM. Statistical significance was determined using one-way ANOVA. **P < 0.001, ***P < 0.001.

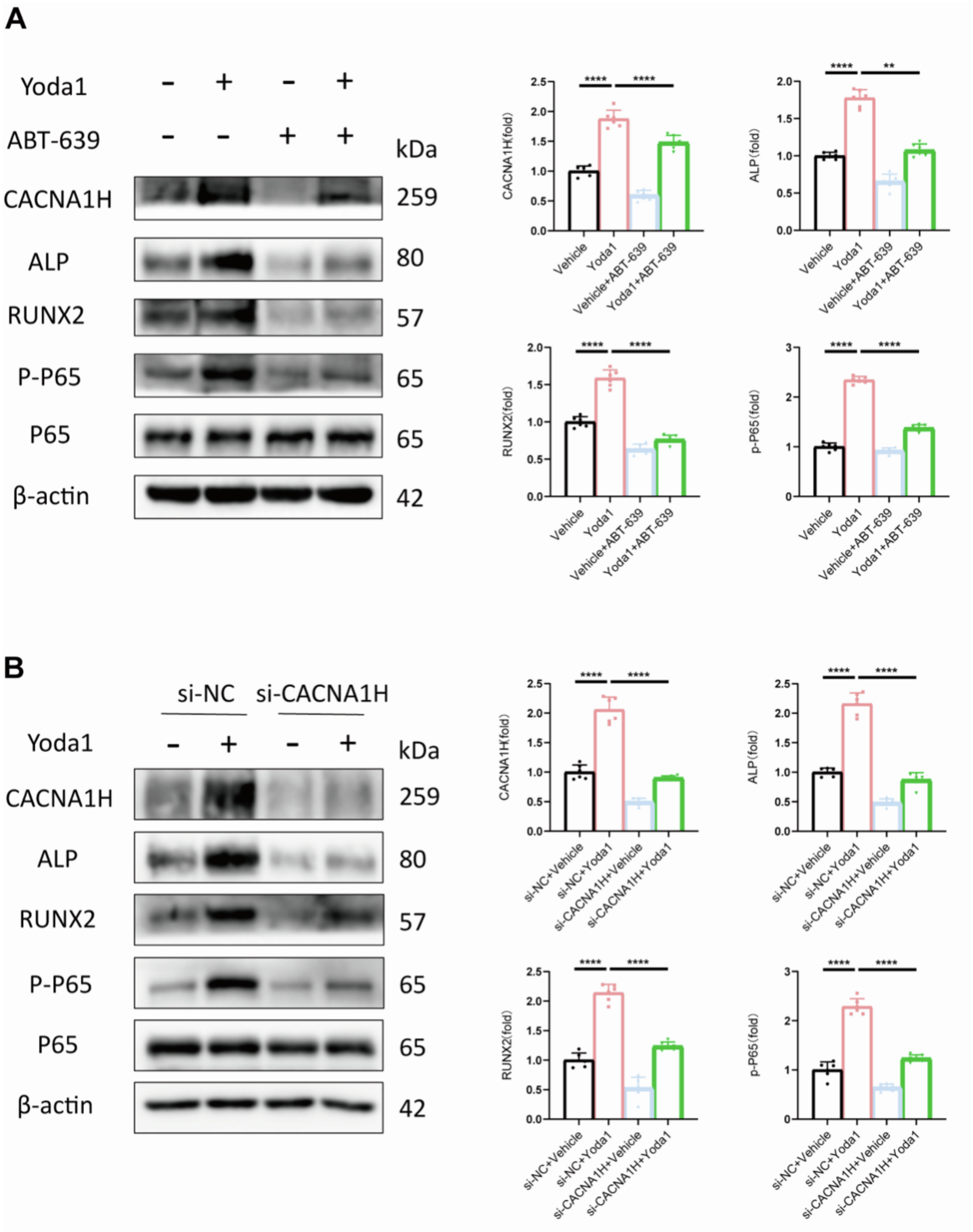

Figure S2. Genetic and pharmacological inhibition of *CACNA1H* attenuates Yoda1-induced pro-calcific responses in VICs. (A) Representative immunoblots and quantitative analysis demonstrating that ABT-639 reduces Yoda1-induced osteogenic (RUNX2, ALP) and inflammatory (p-P65) protein expression (n = 6).(B) Representative immunoblots and quantitative analysis demonstrating that siRNA-mediated knockdown of *CACNA1H* reduces Yoda1-induced expression of the same markers (n = 6). Data are presented as means ± SEM. Statistical significance was determined using one-way ANOVA. **P < 0.001, ****P < 0.0001.

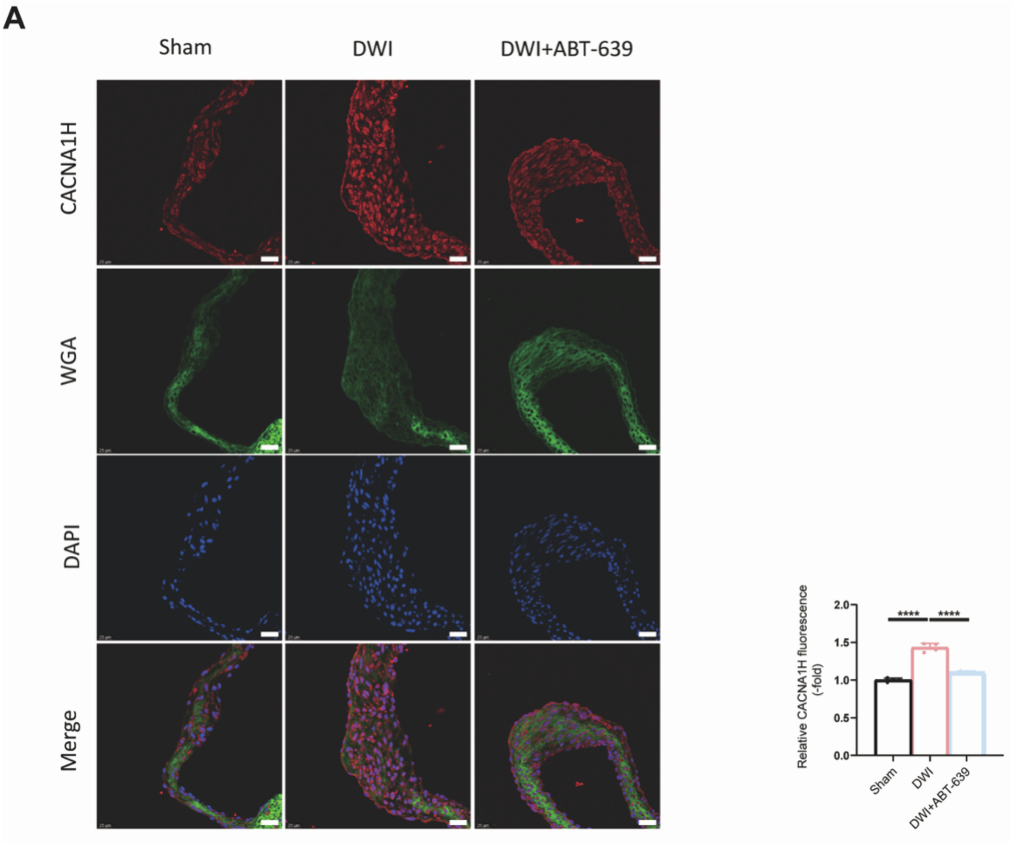

Figure S3. ABT-639 ameliorates DWI-induced upregulation of CACNA1H in vivo. (A) Representative immunofluorescence images of CACNA1H in aortic valves from DWI-treated mice (n = 4, Scale bar = 25 μm). Data are presented as means ± SEM. Statistical significance was determined using one-way ANOVA. ****P < 0.0001.

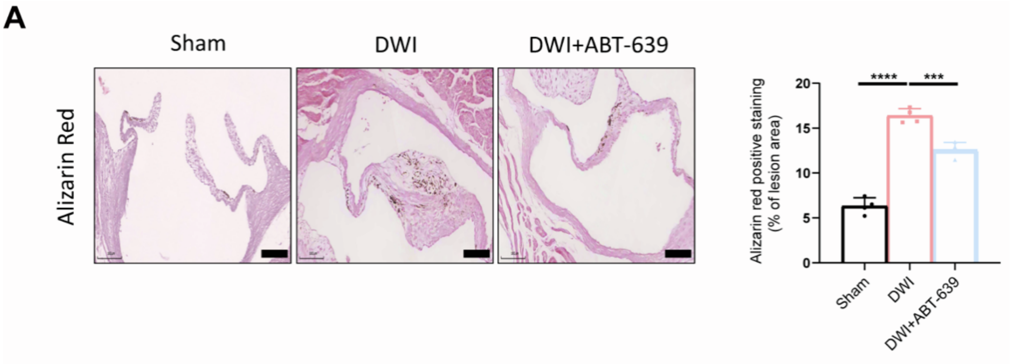

Figure S4. Inhibition of CACNA1H attenuates aortic valve calcification in DWI-treated mice. (A)Representative images of Alizarin red staining in aortic valves from the Sham, DWI, and DWI+ABT-639 groups (n = 4. Scale bar = 100 μm). Data are presented as means ± SEM. Statistical significance was determined using one-way ANOVA. ***P < 0.001, ****P < 0.0001.

Supplementary Table

	Calcific					Healthy Control				
Aortic Valve Stenosis (0=Normal, 1=Calcific)	1	1	1	1	1	0	0	0	0	0
Severity (0=None, 1=Mild, 2=moderate, 3=severe)	3	3	3	3	3	0	0	0	0	0
Regurgitation (1=Yes. 2=None)	1	1	1	1	1	1	0	1	1	0
Vmax (m/s)	6.37	5.24	5.38	4.30	4.23					
ΔPm (mmHg)	94	66	64	40	47					
AvA (cm^2)	0.52	0.43	0.44	0.83	0.82					
LVEF (%)	42.72	67.29	54.15	68.31	68.02	61.54	58.64	56	40.13	63.79
Gender (1=Male, 2=Female)	2	1	2	1	1	1	1	1	1	2
Age (Year)	65	71	85	78	86	68	69	87	68	71
Weight (kg)	57	65	75	65	60	55	68	52	62.5	60
Height (cm)	152	174	150	170	165	160	168	165	165	165
BMI (kg/m^2)	24.0	21.5	33.3	22.5	22.0	21.5	24.1	19.1	23.0	22.0
SBP (mmHg)	126	124	140	125	158	161	142	131	169	135
DBP (mmHg)	74	77	90	76	77	86	84	81	88	88
Smoking (1=Yes, 0=None)	0	1	0	1	1	1	1	0	1	0
CAD (1=Yes, 0=None)	0	0	1	1	1	1	0	0	0	0
Hypertension (1=Yes, 0=None)	0	1	0	1	1	1	1	1	1	1
Diabetes (1=Yes, 0=None)	0	1	1	0	1	0	0	0	0	0

Hyperlipidemia (1=Yes, 0=None)	0	1	0	1	0	1	1	0	0	1
Heart failure (1=Yes, 0=None)	0	0	1	1	0	1	0	1	1	0
Stroke (1=Yes, 0=None)	0	1	1	1	0	0	1	0	0	0
peripheral vascular disease (1=Yes, 0=None)	0	0	1	1	0	0	0	0	0	0
Anti-hypertensive (1=Yes, 0=None)	0	1	0	1	1	1	1	1	1	1
Glucose-lowering (1=Yes, 0=None)	0	1	1	0	1	0	0	1	0	0
Aspirin (1=Yes, 0=None)	0	1	1	1	1	1	0	0	0	0
Statin (1=Yes, 0=None)	1	1	1	1	1	1	1	1	0	1
TC (mmol/L)	3.26	4.21	4.86	5.37	3.4	4.8	7.18	3.31	5.08	5.39
Trig (mmol/L)	0.92	2.72	1.39	3.82	0.94	2.85	2.49	0.52	0.68	1.32
HDL (mmol/L)	1.61	1.21	1.12	0.83	1.46	1.2	1.51	1.44	1.27	1.79
LDL (mmol/L)	1.37	2.62	3.13	3.58	1.58	2.99	4.99	1.75	3.46	3.32
Creatinine (µmol/L)	82	138	82	106	192	116	123	183	189	134
TnT (µg/L)	0.013	0.035	0.108	0.012	0.107	0.02	0.019	0.026	0.039	0.01
NT-ProBNP (pg/mL)	62.39	773	5825	197	5845	2100	24.27	3103	7662	480

Table S1. Baseline characteristics of the aortic valve donors.

Data S1. Original western blotting raw bands.

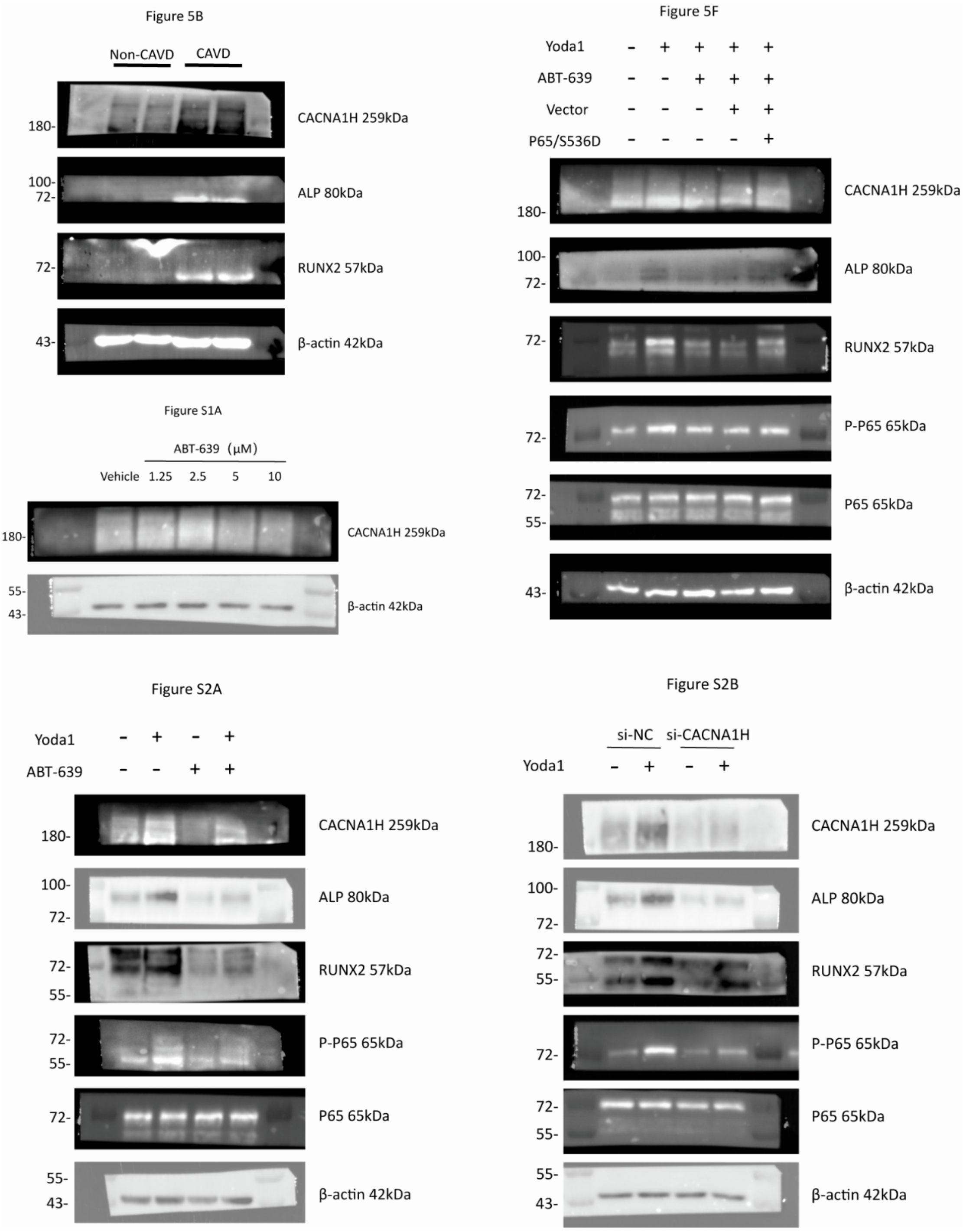

Data S2. Original microscopy images.

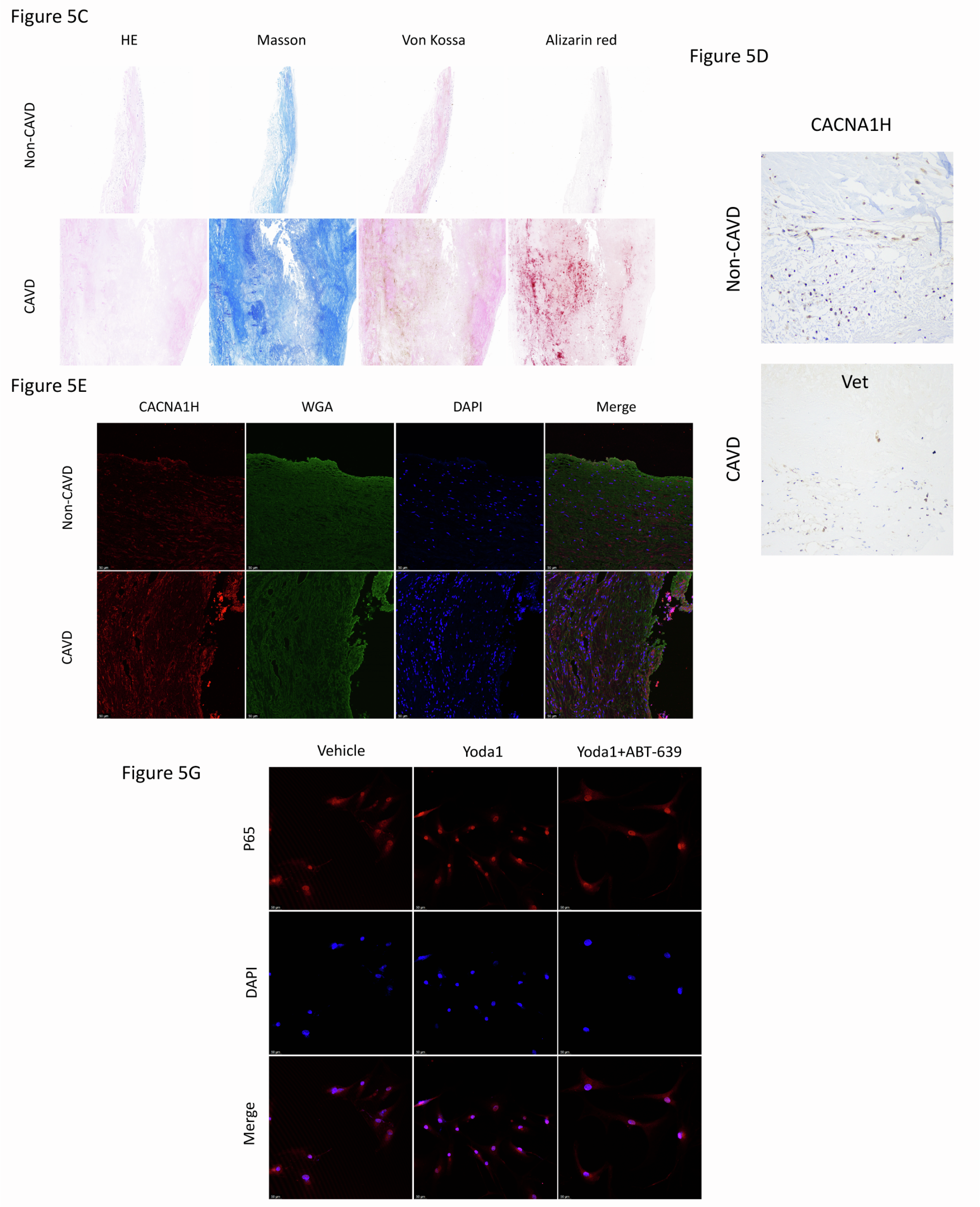

Figure 5I

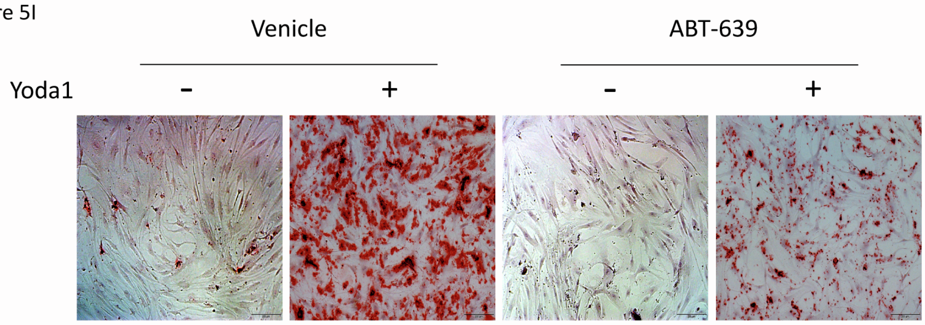

Figure 5J

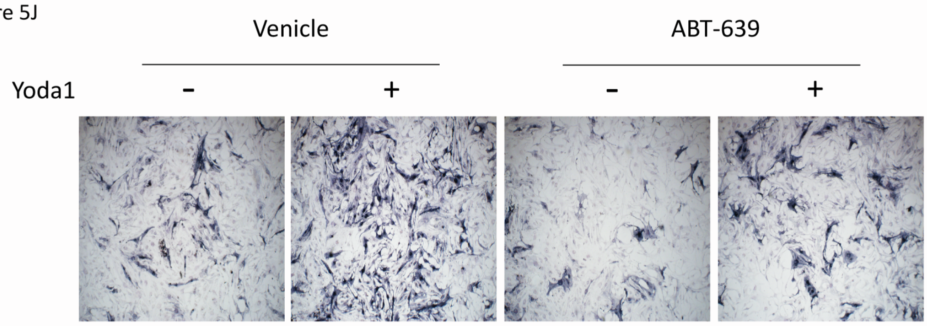

Figure 6B

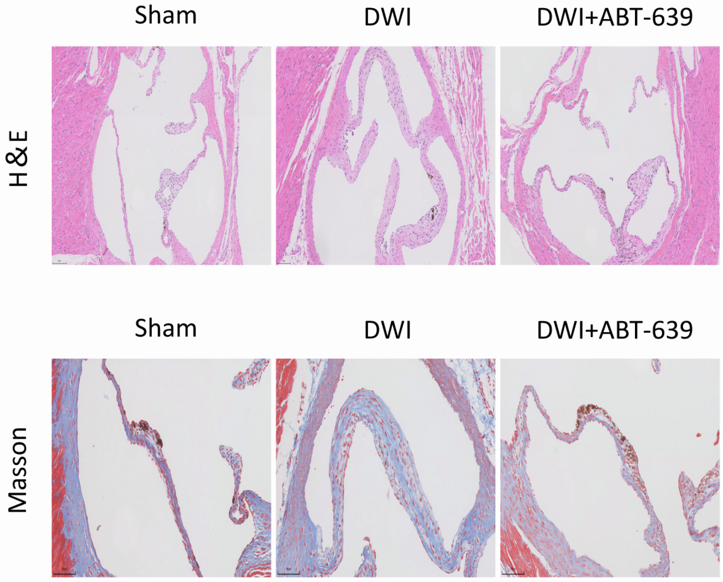

Figure 6D

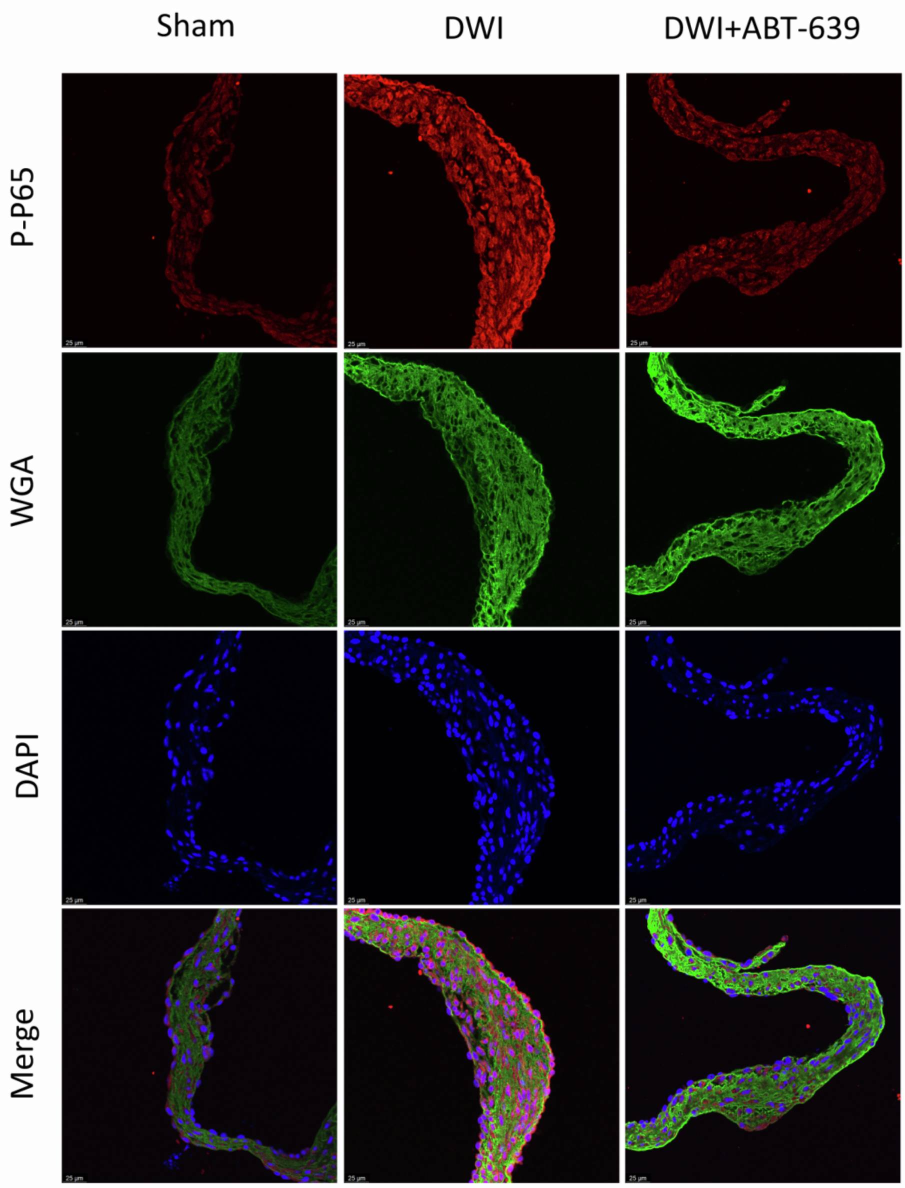

Figure 6E

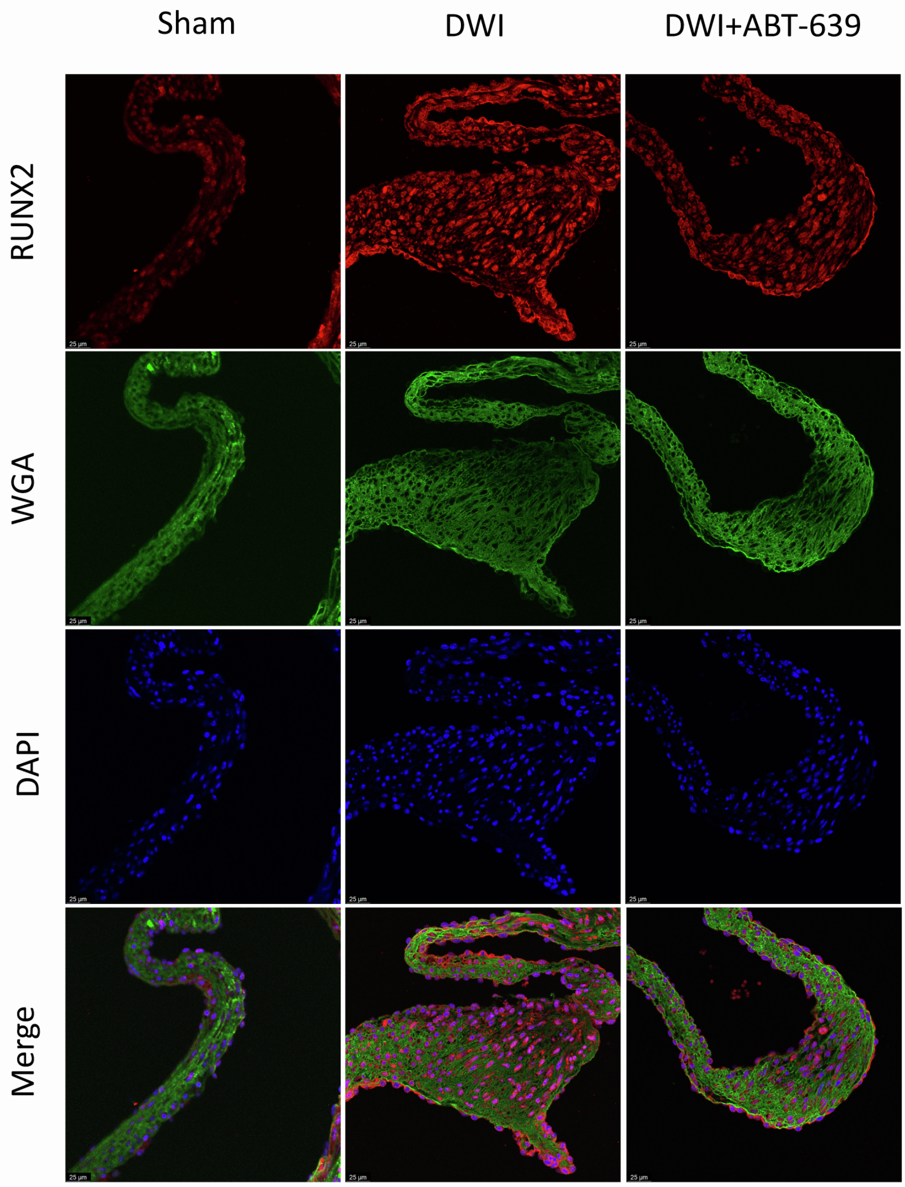

Fig S3A

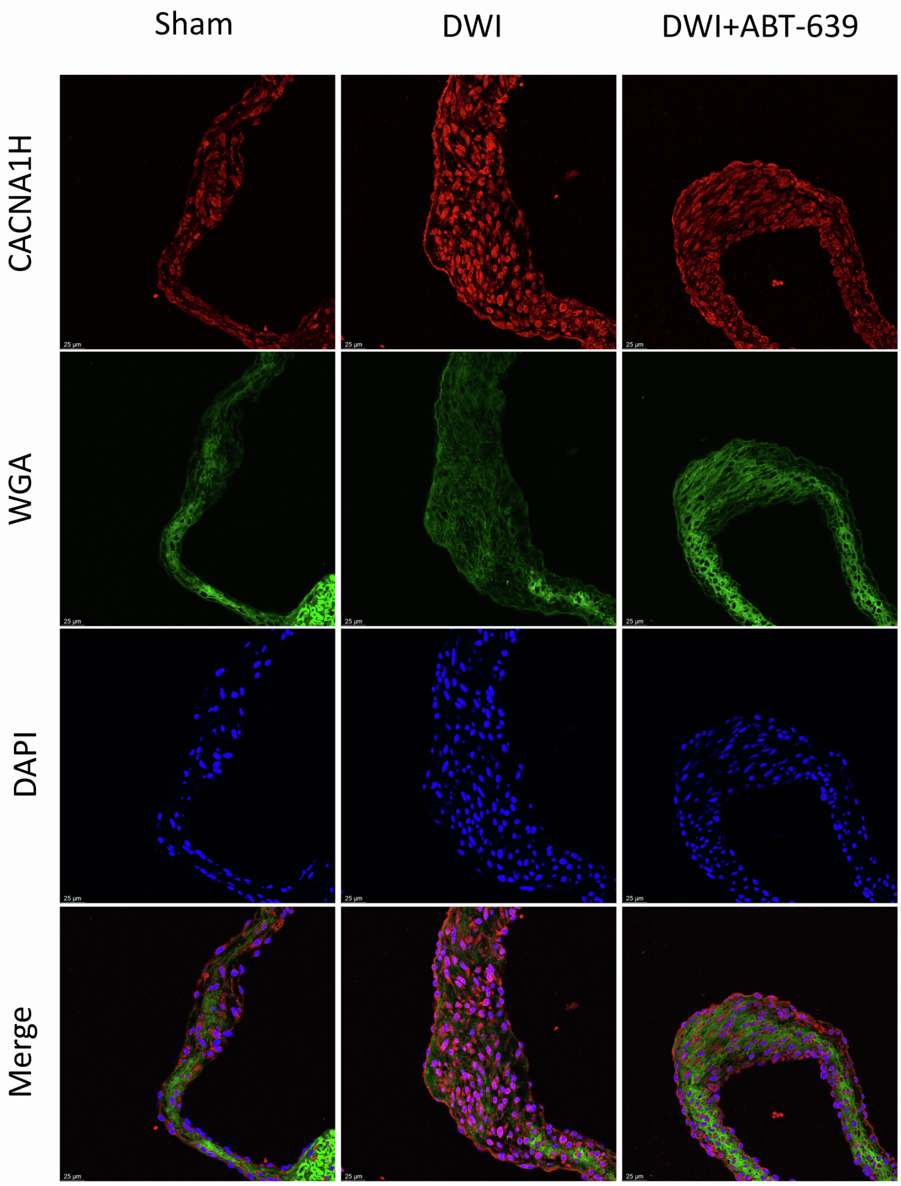

Fig S4A

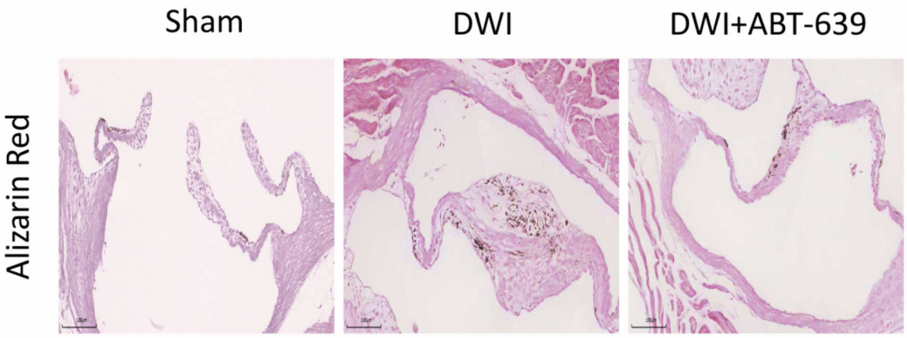

Supplement: Document S1. Figures S1–S4, Table S1, and Data S1–S2 [file mmc1.pdf]
